# The Diverse Oncogenic and Tumor Suppressor Roles of microRNA-105 in Cancer

**DOI:** 10.3389/fonc.2019.00518

**Published:** 2019-06-20

**Authors:** Jie Li, Zhiyuan Zhang, Fangyu Chen, Tao Hu, Wen Peng, Qiou Gu, Yueming Sun

**Affiliations:** ^1^First Clinical Medical College, Nanjing Medical University, Nanjing, China; ^2^Department of Colorectal Surgery, The First Affiliated Hospital of Nanjing Medical University, Nanjing, China; ^3^Department of Radiation Oncology, The First Affiliated Hospital of Nanjing Medical University, Nanjing, China

**Keywords:** miR-105, cancer, oncogene, tumor suppressor, exosome, clinical implication

## Abstract

MicroRNAs (miRNAs) are non-coding small RNA molecules that regulate gene expression at the post-transcriptional/translational level. They act a considerable role not only in the normal progress of development but also in aberrant human diseases, including malignancy. With accumulating proofs of miR-105, the complex role of miR-105 during cancer initiation and progression is gradually emerging. miR-105 acts as a tumor suppressor by inhibiting tumor growth and metastasis or as an oncogene by promoting tumor initiation and invasion, depending on particular tumor contexts and base-pairing genes. In this review, we emphasize the characteristics of miR-105 in cancer to elucidate various deadly tumors and discuss transcriptional regulations that may explain fluctuations in miR-105 expression. This review may provide new ideas for applying miR-105 as a diagnostic and prognostic biomarker.

## Introduction

**Figure 1:**
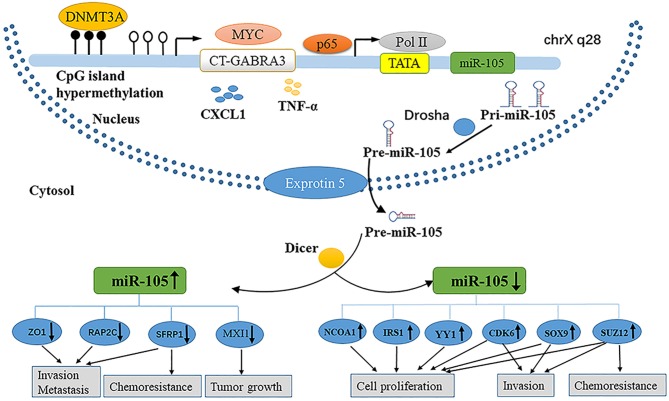
Diverse roles and regulation of miR-105 in cancer.

MicroRNAs (miRNAs) are small (~22 nt) non-coding RNAs that regulate gene expression through base-pairing with the 3′ untranslated regions (UTRs) of target messenger RNAs (mRNAs) (1). Similar to protein-coding genes, miRNAs are produced by endogenously transcribed long primary transcripts (pri-miRNAs), which are further cleaved into pre-miRNAs (2) (Figure 1).

Then, the pre-miRNAs are exported from the nuclei by double-stranded RNA-binding proteins (exportin-5) and then cleaved by RNase III family enzymes (Dicer) into incompletely mature miRNAs (3–5). The mature miRNA is incorporated into the RNA-induced silenced complex (RISC) (6, 7). The active RISC complex then binds to a target mRNA with a complementary sequence to trigger the mRNA for subsequent silencing (8). miRNA genes are involved in the regulation of up to 30% of all protein-coding genes (9, 10). Although the scope of miRNA regulation of the human transcriptome is still under investigation, the association of miRNA dysregulation with the occurrence and development of various diseases has been supported by evidence (11).

The miRNA-105 family, which consists of three members (miR-105-1, miR-105-2, and miR-767), is located on human chromosome Xq28. The first report showing the relationship between miR-105 and human health demonstrated its downregulation in the primary myelofibrosis granulocytes in comparison with that in normal subjects (12). Afterward, genome-wide miRNA expression profiling studies exhibited that miR-105 is widely expressed in various tissues or organs and is significantly aberrant in malignant cells such as in colorectal cancer (CRC) (13, 14), hepatocellular carcinoma (HCC) (15, 16), gastric cancer (GC) (17, 18), breast cancer (BC) (19–21), lung cancer (22, 23), and glioma (24–28) (Table 1).

**Table 1:** The expression, targets, biological functions and role of miR-105 in cancer.

**Cancer type**	**Expression**	**Site (tissue/plasma/cell)**	**Targets**	**Biological functions**	**Role**	**References**
Colorectal cancer	Up	Tissue and cell line	RAP2C	Promote cell metastasis and EMT	Tumor promoter	(13, 14)
Gastric cancer	Up Down	Tissue Tissue	_ YY1	_ Inhibit cell viability and proliferation	_ Tumor suppressor	(17) (18)
Triple-negative breast cancer	Up	Tissue and plasma	SFPR1	Promote cell stemness, chemoresistance, and metastasis	Tumor promoter	(21)
Glioma	Down	Tissue and cell	SOX9 and SUZ 12	Represses cell proliferation, tumorigenesis, drug sensitivity and invasion and induces apoptosis	Tumor suppressor	(24–28)
Hepatocellular carcinoma	Down	Tissue and cell	NCOA1, IRS1, PDK1 and AKT1	Inhibit cell proliferation and tumorigenicity	Tumor suppressor	(15, 16)
Prostate cancer	Down	Cell	CDK6	Inhibit cell proliferation and invasion	Tumor suppressor	(29)
Non-small-cell lung cancer	Down	Tissue	_	_	_	(22)
	_	_	Mcl-1	Promote cell viability, migration and EMT	Tumor promoter	(23)
Breast cancer	Up	EVs and plasma	ZO-1 and MXI1	Promote cell metastasis and tumor growth	Tumor promoter	(19, 20)

However, contradictory results were produced through previous research with regard to whether miR-105 is an oncogene or a tumor suppressor and whether it is a positive or negative prognostic biomarker. In this review, we examine the following according to the properties of miR-105: (1) the oncogenic roles of miR-105, (2) miR-105 as a tumor suppressor, (3) the functions miR-05 in cancer-cell-secreted exosome, (4) the regulation mechanisms explaining the aberrant expression of miR-105, and (5) the diagnostic and prognostic value of miR-105 in cancer. The aim of this review is to emphasize the novel and diverse functions of miR-105 and its sense in cancer therapy.

## Oncogenic Roles of miR-105

### Colorectal Cancer

The vital roles of miRNAs, especially miR-105, in the pathophysiology of colorectal cancer (CRC) have been demonstrated in various studies (30–32). Through high-throughput sequencing, Hamfjord et al. found that the level of miR-105 expression was upregulated in tumor tissues from eight patients with CRC. Further data confirmed the differences in miRNA expression between adenocarcinomas and neuroendocrine of colon cancer (13). Shen et al. also reported that the miR-105 is overexpressed in CRC tissues and cell lines, and miR-105 expression positively correlates with lymph node invasion, TNM stage, and metastasis. Moreover, miR-105 expression is predominantly stimulated by TNF-α in a time-dependent manner. Upregulated miR-105 expression promotes CRC cell metastasis and epithelial-mesenchymal transition (EMT) *in vitro* and *in vivo* by directly targeting the 3′UTRs of a Rap2 subfamily of a small GTP-binding protein (RAP2C) through the activation of the NF-κB signaling pathway (14). Thus, miR-105 has an oncogenic role in human CRC. However, the abovementioned researches are relatively superficial, and future studies are needed to confirm the specific mechanism of miR-105 in CRC.

### Gastric Cancer

Although several miRNAs have demonstrated their crucial roles in the occurrence and progression of gastric cancer (GC) (33–35), the exact role of miR-105 in GC remains unclear (17, 18). Liu et al. first reported that miR-105 expression is significantly elevated in GC tissues than in normal tissues (17). However, Zhou et al. recently revealed that a highly methylated miR-105 promoter decreases miR-105 expression in GC tissues (18). Overexpressed miR-105 inhibits cell viability and proliferation by directly targeting the Yin Yang 1 (YY1). The miR-105 role in GC is complex and thus must be investigated further.

### Triple-Negative Breast Cancer

miR-105 functions as a tumor promoter by targeting corresponding genes in BC (19–21). Li et al. reported that miR-105 is upregulated specifically in the cancer tissues of patients with triple-negative breast cancer (TNBC). Currently, the treatment of TNBC is very disappointing (36). High miR-105 expression correlated with poor survival in patients with TNBC, and miR-105 promoted the stemness, chemoresistance, and metastasis of the TNBC cells by activating Wnt/β-catenin signaling by targeting SFPR1. Moreover, circulating miR-105 levels were significantly elevated in the plasmas of 74 patients with TNBC relative to the miR-105 levels of 44 patients without TNBC or 12 healthy controls, indicating that the circulating miR-105 may serve as a powerful biomarker for TNBC (21).

## miR-105 as a Tumor Suppressor

### Glioma

Yan et al. investigated that miR-105 is significantly lower in anaplastic gliomas and secondary glioblastomas than in low-grade gliomas. High miR-105 levels are commonly related to favorable prognosis in patients with glioma (25), and miR-105 level is reduced in glioblastoma unlike in low-grade gliomas (28). Guan et al. found that gliomas show larger decrease in miR-105 expression than non-neoplastic brains. Furthermore, low miR-105 expression is statistically associated with advanced tumor grade and unfavorable clinical outcome (24). Liu et al. reported that miR-105 is commonly downregulated in glioma tissues and cells and the level of miR-105 expression decreases with increasing tumor stage. Gain and loss-of-function experiments indicated that miR-105 upregulation represses proliferation and invasion and induces apoptosis by directly targeting the mRNA of SOX9 and inhibiting the downstream TCF4, c-MYC, Cyclin D1, and AXIN2 protein expression (27). In another study, Zhang et al. confirmed that miR-105 level is remarkably reduced in glioma tissues and cell lines and ectopic miR-105 inhibits cell proliferation, tumorigenesis, metastasis, and drug sensitivity by downregulating the suppressor of Zeste 12 in glioma (26). The abovementioned researches suggest that targeting miR-105 and its downstream factors is a promising therapeutic strategy for glioma treatment.

### Hepatocellular Carcinoma

To date, human endogenous miRNA dysregulation has been involved in the carcinogenesis and progression of HCC (37–39). Shen et al. found that HCC cell lines and clinical HCC tissues show significant decrease in their miR-105 expression levels in contrast to normal hepatocytes and adjacent non-cancerous tissues. Decreased miR-105 expression promotes the proliferation and tumorigenicity of HCC cells *in vitro* and *in vivo* and activates the phosphoinositide 3-kinase (PI3K)/AKT signaling pathway by directly upregulating insulin receptor substrate-1, 3-phosphoinositide-dependent protein kinase-1, and AKT1, leading to decreased cyclin-dependent kinase inhibitors of 1A and 1B (p21^Cip1^ and p27^Kip1^) and increased cyclin D1 expression in HCC (16). Another study showed that miR-105 expression was reduced in HCC tissues (15). The upregulated expression of miR-105 suppresses the proliferation of HCC cells *in vitro* by targeting the nuclear receptor coactivator 1 (NCOA1). Furthermore, decreased miR-105 correlates with reduced median OS and PFS in patients with HCC. The research on the mechanism of miR-105 loss will help future scholars better understand the tumor transformation in HCC and design new treatment strategies.

### Other Tumors

Apart from the aforementioned tumors, the miR-105 as a tumor suppressor has been widely studied in other sort of cancer, including prostate cancer and non-small-cell lung cancer (NSCLC). Honeywell et al. reported that prostate cancer cell lines have downregulated miR-105 expression compared with normal prostate epithelial cells. miR-105 upregulation inhibits tumor cell proliferation and invasion *in vitro* and tumor growth *in vivo*, and CDK6 is deemed as a new target of miR-105 (29). Consistently, Lu et al. also reported that the level of miR-105-1 is decreased in NSCLC tissues and that a decreased miR-105-1 level is associated with a large tumor size and poor overall survival (OS) and disease-free survival of patients with NSCLC (22). Interestingly, Jin et al. found that an elevated miR-105 significantly enhances the viability and migration of NSCLC cells *in vitro* through activating the mTOR and p38MAPK pathways by targeting Mcl1 (23). However, further researches are needed to fully characterize the biological function of miR-105 in these cancers.

## Exosomal miR-105 by Cancer-Cell Secretion

**Figure 2:**
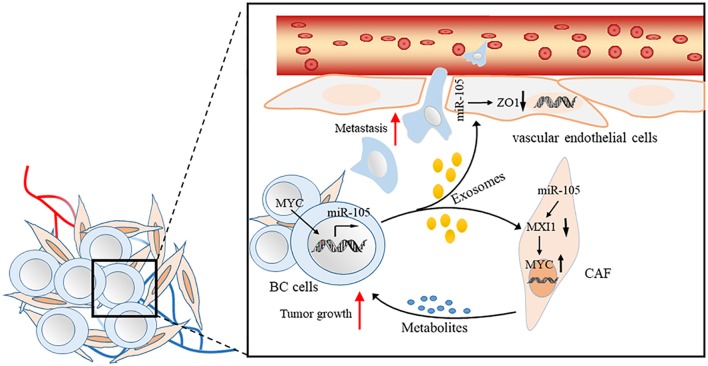
Proposed model of promoting BC cell metastasis and tumor growth by cancer-secreted EVs through a miR-105-mediated mechanism identified herein.

Exosomes are small (50–100 nm) extracellular vesicles (EVs) with lipid bilayer membrane, whose release are conducted by fusion with the cell membrane (40–42). Exosomes have been identified as key factors mediating interactions between tumor cells and other stromal cells in tumor microenvironment. Exosomal miRNAs secreted by tumor cells can enter other non-cancer cells by endocytosis (40, 42–45). Cancer-secreted miRNAs act as an essential role in facilitating the cancer initiation and progression (46–49). Zhou et al. first found that metastatic BC (MBC) cells, which secrete exosomal RNAs, significantly stimulate the migration efficiency of human microvascular endothelial cells (HMVECs). The level of miR-105 expression is markedly higher in exosomes derived from MBC cells than in non-cancerous mammary epithelial cells. Meanwhile, the ectopic cancer-derived miR-105 is transferred via exosomes, thereby dramatically reducing ZO-1 expression and breaking the barrier function of HMVECs that promote metastasis both *in vitro* and *in vivo* (19). Yan et al. found that the uptake of EVs from BC cells by patient-derived cancer-associated fibroblasts (CAFs) induces the enrichment of a gene signature related to MYC activation in CAFs compared with non-cancerous mammary epithelial cell EVs or phosphate buffer saline by gene set enrichment analysis. In search of the potential upstream event leading to MYC activation, they observed MAX-interacting protein 1 (MXI1), which is a well-known antagonist of MYC transcriptional activity, is downregulated by >50% (50, 51). Moreover, miR-105 directly targets MXI1 in which its expression level is greatly higher in EVs derived from BC cells than in non-cancerous mammary epithelial cells. The abnormal expression of miR-105 in EV is the result of the MYC oncoprotein in BC cells and in turn, acts on MXI1 in CAFs and activates MYC signaling to induce a metabolic program that is suitable for tumor growth. Thus, the MYC-miR-105-MXI1-MYC-signaling axis-mediated metabolic reprogramming of stromal cells contributes to a sustained tumor growth by conditioning the shared metabolic environment (20) (Figure 2).

miR-105 is present in serum-derived exosomes, and the level of miR-105 expression in patients with distant metastasis is higher than in patients who did not develop metastasis (19). These studies provide insights into the regulation of gene expression and cellular components of the tumor microenvironment by exosomally derived miRNAs and indicate that miR-105 has potential clinical applications as a prognostic biomarker and a therapeutic target for BC.

## Regulation Mechanisms of miR-105 Expression

Parallel to protein-encoding genes, the expression of miRNA is regulated not only by different mechanisms at a genetic or epigenetic level but also by the dysregulation of specific transcription factors (52). Similarly, the dysregulation of miR-105 in cancer is involved in some of the aforementioned mechanisms (Figure 1).

Epigenetic regulation is an important mechanism governing miRNA expression (53, 54). Zhou et al. reported that the expression of miR-105 is downregulated concomitantly with the DNA hypermethylation of CpGs in the upstream region of the miR-105 promoter in GC. Furthermore, the knockdown of DNA methyltransferase (DNMT3A) could regain the level of miR-105 expression, which is suppressed in GC (18). Meanwhile, Loriot et al. demonstrated that the hypomethylation of CpGs in the upstream region of the miR-105 promoter activates the cancer-germline transcript (CT-GABRA3) that inducess the high expression of miR-105 and miR-767 in melanoma, immortalized fibroblast, and embryonal carcinoma cell lines (55). Through chromatin immunoprecipitation (ChIP) assays, Ji et al. confirmed that the NF-κB transcription complex p65 binds to the upstream promoter regions of miR-105 genes to silence transcription; p65 is recruited beforehand by the fibroblast growth factor 2 (FGF2), and miR-105 expression is restored by suppressing p65 in human osteoarthritis chondrocytes (56). Yan et al. found that the abnormal expression of MYC upregulates miR-105 expression both in the cells and in EVs, while MYC knockdown downregulates intracellularly and secretes miR-105 in BC cells expressing high miR-105 levels. Then, a thorough search of the promoter of GABRA3, the hosting gene harboring hsa-mir-105-1/2, revealed the presence of an E-box which responded to MYC to activate reporter gene expression (20). The alteration of the tumor microenvironment has a novel mechanism of the miRNA expression (57). Shen et al. found that the level of miR-105 expression is markedly stimulated by TNF-α in a time-dependent manner through employing RT-qPCR analysis in CRC cells (14). By next-generation sequencing and RT-qPCR, Hsu et al. found that miR-105 expression is upregulated in cells that is treated with CXCL1 secreted from tumor-associated dendritic cells in colon cancer (58).

## Clinical Implications

Improving the therapeutic result with the early diagnosis and accurate prognosis evaluation of cancer is critical. Cancer cells or tissues may present aberrant miRNA expression profiles, and specific miRNA features can be used not only for diagnosis but also for classifying patients with cancer as subgroups with different prognosis for individualized treatment (59–63). The role of miR-105 in cancer diagnosis and prognosis is extensively investigated, but it exhibits apparently conflicting outcomes (Table 2).

**Table 2:** Summary of clinical studies investigating the role of miR-105.

**Cancer types**	**miR-105 expression**	**Sources**	**Sample number**	**Prognosis or outcome of low miR-105**	**References**
BC	Up	Serum	38	Good	(19)
Hepatocellular cancer	Down	Tissue	188	Poor	(15)
Gliomas	Down	Tissue	76	Poor	(24)
Gliomas	Down	Tissue	116	Poor	(25)
TNBC	Up	Serum/tissue	86/13	Good	(21)
NSCLC	Down	tissue	174	Poor	(22)

Zhou et al. demonstrated that patients who later developed distant metastases have higher levels of circulating (exosomal) and tumor miR-105 than patients who did not and had normal mammary tissues, suggesting that cancer-derived miR-105 can serve as a blood-based marker for the early diagnosis of BC metastasis (19). High NCOA1 and low miR-105-1 levels significantly decrease OS (*P* < 0.001) and PFS (*P* = 0.002), implying that NCOA1 and miR-105-1 might have a potential prognostic value for patients with HCC (15). Guan et al. found that a decreased miR-105 expression is statistically associated with advanced clinical features and poor OS (P < 0.001) of patients with glioma, suggesting that miR-105 downregulation may be used as a malignant prognostic marker in gliomas (24). Similarly, Yan et al. indicated that low miR-105 expression can be used for identifying patients with a high risk of unfavorable outcomes, particularly as a prognostic marker for patient risk stratification in anaplastic gliomas and secondary and proneural glioblastomas (25). A high level of miR-105 is associated with advanced clinical stages and the increased rates of the distant metastasis of CRC (14). Li et al. found that circulating miR-105/93-3p can act as an early diagnostic biomarker for TNBC, and the levels of miR-105/93-3p are significantly elevated in the plasmas of patients with TNBC relative to the levels in the plasmas of patients without TNBC or healthy controls. As expected, the patients with high miR-105/93-3p levels in TNBC tissues showed a positive correlation with chemoresistance and poor survival (21). Reduced miR-105-1 expression is associated with a larger tumor size, as well as the poor OS and disease-free survival of patients with NSCLC, demonstrating that downregulated miR-105-1 can be used as an independent malignant predictor in patients with NSCLC (22).

## Conclusion

miRNAs play a vital role in the initiation and progression of human malignancies. On the one hand, miR-105 facilitates cell proliferation, promotes metastasis and chemoresistance, and initiates EMT in tumorigenesis, as illustrated in Figure 1. On the other hand, miR-105 inhibits proliferation, tumorigenesis, migration, invasion, and drug sensitivity. The role of this miRNA depends on the cellular and histological features of tumors and target mRNAs. The fluctuation of miR-105 expression is correlated with epigenetic alterations, especially the regression of miR-105 in cancer. Nevertheless, further researches are needed to provide sufficient evidence to explain the activation of miR-105 in cancer.

## Author Contributions

JL, ZZ, and FC contributed to conception and manuscript writing. TH, WP, and QG searched the literature. YS participated in its coordination and modification. All the authors have read and approved the final manuscript.

### Conflict of Interest Statement

The authors declare that the research was conducted in the absence of any commercial or financial relationships that could be construed as a potential conflict of interest.
